# Foliar Nutrition, Biostimulants and Prime-Like Dynamics in Fruit Tree Physiology: New Insights on an Old Topic

**DOI:** 10.3389/fpls.2017.00075

**Published:** 2017-02-01

**Authors:** Georgia Tanou, Vasileios Ziogas, Athanassios Molassiotis

**Affiliations:** Laboratory of Pomology, Faculty of Agriculture, Aristotle University of ThessalonikiThessaloniki, Greece

**Keywords:** abiotic stress, fruit tree nutrition, priming, reactive nitrogen species, reactive oxygen species, reactive sulfur species

## Abstract

Despite the fact that the usage of foliar nutrients has long history, many aspects of fertilization through leaves are still unknown. Herein, we review the current knowledge regarding the canopy fertilization putting special emphasis on Fe nutrition and briefly provide insights into the nanofertilizer technology of the foliar feeding of fruit crops. In addition, this paper discusses the main aspects of the foliar application of biostimulants regarding crucial factors of fruit cropping systems, such as fruit yield/size, tolerance to environmental stresses, and nutrient availability. Also, we specifically discuss the role of hydrogen peroxide (H_2_O_2_) and nitric oxide (NO) as priming molecules and their possible cross-talk with biostimulants in fruit tree physiology. Finally, a view of the key issues for future fundamental and applied research in the topic is put forward.

## Introduction

Fruit tree crops are agricultural commodities of great biological and economical importance, and therefore, precise knowledge of treatments that boost fruit production, is of great importance. It is generally accepted that the appropriate nutrient management is crucial for optimizing fruit crop production. However, fruit growers usually apply larger amounts of chemical fertilizers to the soil than the tree actually needs, resulting to surface runoff and environmental pollution (Vance, 2001). Foliar sprays have been also used as an important tool to meet tree nutrient demand. This fertilization method is more target-oriented and environmental friendly since the nutrients are applied in controlled quantities (Fernández and Eichert, 2009).

Perennial fruit trees are frequently exposed to various abiotic stresses during their lifetime that limit crop yield. To face this problem, modern fruit tree physiology is currently focused, among others, on the stimulation of stress-related tolerance mechanisms and plant cell development programs using biostimulants. Biostimulants are incorporated in the practice of fruit production due to their ability to enhance nutrient uptake, stimulate plant development and minimize the use of fertilizers (Kunicki et al., 2010). Despite the widespread use of biostimulators in fruit industry, their precise mechanisms of action remain unknown. On the other hand, the use of priming techniques (e.g., external application of natural or synthetic compounds in plants to induce acclimation against environmental stresses) has also received much attention in recent years (Tanou et al., 2012b). The priming (also called hardening) is a process by which plants attain a unique physiological state called “primed” state, after a pre-treatment with a given priming agent. Detailed study of the molecular aspects of priming in plants, including fruit crops has recently been reviewed elsewhere (Conrath, 2011; Tanou et al., 2012b; Molassiotis et al., 2016).

This review is divided into three parts. The first provides a short overview of foliar fertilization in fruit tree physiology. The second part covers the biostimulants effects and possible physiological mechanisms, while the last part refers to tree responses to hydrogen peroxide (H_2_O_2_) and nitric oxide (NO) priming agents. Finally, the interplay between biostimulants and H_2_O_2_- and NO-associated priming in whole-tree physiology is also addressed.

## Canopy fertilization in fruit trees in general: Fe deficiency as an example

Taking into account the limitations of nutrients addition through soil, foliar sprays are an effective way to meet the plants nutrients requirements (Wójcik, 2004). The most common macronutrients applied as foliar fertilizers are N (as urea, ammonium nitrate and ammonium sulfate), P [as H_3_PO_4_, KH_2_PO_4_, NH_4_H_2_PO_4_, Ca(H_2_PO_4_)_2_ and phosphites], K (as K_2_SO_4_, KCl, KNO_3_, K_2_CO_3_, KH_2_PO_4_), Mg (as MgSO_4_, MgCl_2_, Mg(NO_3_)]_2_, and Ca (as CaCl_2_, Ca-propionate, Ca-acetate). Also, to the most commonly foliar-applied micronutrients belong the B [as boric acid (B(OH)_3_], borax (Na_2_B_4_O_7_), Na-octoborate (Na_2_B_8_O_13_), B-polyols, Fe [as FeSO_4_, Fe(III)-chelates, Fe-complexes], Mn [as MnSO_4_, Mn(II)-chelates], and Zn [as ZnSO_4_, Zn(II)-chelates, ZnO, Zn-organic ‘complexes] (Fernández et al., 2013). Historically, research on foliar fertilization was started at the late 1940s and early1950s. Later, extensive foliar fertilization research was conducted for high-value fruit crops challenged with microelement deficiencies. Recently, a considerable amount of new information has been gathered regarding the absorption, translocation, and utilization of foliar-applied nutrients by fruit trees (Fernández and Brown, 2013). Given that the penetration of the cuticle is generally considered to be the rate-limiting step for foliar nutrition, several hypotheses about the penetration of nutrient via the cuticle have been raised (Fernández et al., 2016). For instance, there is evidence, although still not extensive, that polar paths of diffusion across cuticles exist (Niemann et al., 2013). Ionic compounds use aqueous polar paths of diffusion, whereas lipophilic molecules diffuse along the lipophilic wax and cutin domains (Schreiber, 2005). The nature of these polar domains remains to be explained in more detail.

In many orchards, macronutrients and especially micronutrients, such as B, Zn, Mn, and Fe were leaf-applied regularly to prevent the deficiencies of these elements. Particularly, Fe chlorosis is recognized as a suitable model to study foliar fertilization since it represents a common nutritional problem in several fruit crops, such as citrus, pear, and peach trees, grown on calcareous soils (Molassiotis et al., 2006). Correction of Fe chlorosis is generally carried out by soil application of synthetic Fe(III) chelates, which are usually quite effective. Iron foliar fertilization is a cheaper and more environmentally-friendly alternative to soil treatments with synthetic Fe(III) chelates for the control of Fe chlorosis in trees (El-Jendoubi et al., 2011). Hence, a great deal of additional work is required to understand leaf Fe uptake as well as the characteristics of Fe fertilizers. Using X-ray emission, El-Jendoubi et al. (2014) showed that foliar Fe-sulfate fertilization in Fe-deficient peach leaves were minor outside the leaf surface, indicating that Fe mobility within the leaf is a major constraint for fertilizer effectiveness in fruit crops. Recently, Rios et al. (2016) showed that Fe applied as inorganic salts was absorbed rapidly through the stomata of Prunus rootstock GF 677; strong labile Fe pools stained with blue Perls were detected in vascular areas of the leaf blade and the central vein in response to FeSO, whereas in the case of Fe(III) salts the stain remained at the stomatal area. These results give the possibility to test new Fe fertilizer formulations easily, as well as to study the possible Fe transporters responsible for leaf Fe uptake.

It is noteworthy that, in parallel to the studies on the role of essential nutrients, the function of the beneficial elements on fruit physiology was recently investigated. For instance, the potential effect of leaf-applied titanium (Ti) and silicon (Si) in apple and sapota tree vigor and yield has been proposed (Wójcik et al., 2010; Thippeshappa et al., 2014). In addition to this, it has become apparent that foliar nutrients can regulate flowering, fruit yield and fruit quality. For example, several studies revealed that foliar sprays of B increase pollen-tube germination and fruit set in a number of tree species (Wang et al., 2015).

## Nanotechnology can boost foliar nutrition practices

Nanotechnology is a multidisciplinary and rapidly growing field in science and technology, which involves the manufacture, processing and application of nanometer scale assemblies of atoms and molecules. Nanomaterials are classified as materials with at least one dimension less than 100 nm (Sekhon, 2014). The most important application of nanotechnology in agricultural crop production is the field of nano-fertilizers. Nanofertilizers are nutrient carriers of nano-dimensions capable of binding nutrient ions due to their high surface area and release it slowly and steadily that commensurate with crop demand (Subramanian et al., 2015). In nanofertilizers, nutrients can be encapsulated by nanomaterials, coated with a thin protective film, or delivered as emulsions or nanoparticles. The smaller size, the higher specific surface area and the reactivity of nanofertilizers may affect nutrient solubility, diffusion and hence availability to plants (Singh et al., 2013). Nanofertilizer technology is very innovative, and scant reported literature is available concerning fruit trees. In this context, Davarpanah et al. (2016) indicated that the foliar application of nano-Zn and nano-B fertilizers in pomegranate increased the leaf concentrations of both microelements, reflecting the improvements in tree nutrient status. Thus, more detailed and comprehensive work is needed in this important area of research.

## Agricultural biostimulants in brief: definition, categories, and mode of action

Although the term “biostimulant” has been used for many years, it is still not fully defined. According to European Biostimulant Industry Council (EBIC) “plant biostimulants contain substance(s) and/or micro-organisms whose function when applied to plants or the rhizosphere is to stimulate natural processes to enhance/benefit nutrient uptake, nutrient efficiency, tolerance to abiotic stress, and crop quality” (http://www.biostimulants.eu). The review of the relevant literature reveals a wide range of compounds, including humic and fulvic acids, protein hydrolysates and other N-containing compounds, seaweed extracts and botanicals, chitosan and other biopolymers, beneficial fungi and bacteria potentially act as a biostimulant (du Jardin, 2015). Several studies documented that biostimulants promote plant growth, development and productivity (Brown and Saa, 2015; Bulgari et al., 2015), however the mechanism of action is poorly or not understood. It is possible that the beneficial effects of biostimulant on growth parameters could be ascribed to auxin and gibberellin-like activity, and enhanced nitrogen uptake, as documented for the biostimulant action of plant-derived protein hydrolysate in corn, tomato, and gibberellin-deficient dwarf pea plants (Colla et al., 2014). Another suggested function of biostimulants was linked to reactive oxygen/nitrogen species and hormonal signaling. For example, chitosan, a natural biopolymer produced from chitin, is the major constituent of arthropods exoskeleton and fungi cell walls and has been extensively studied as an elicitor for inhibiting postharvest senescence and diseases in many fruit, such as apple, citrus, kiwifruit, peach, pear, strawberry, and sweet cherry (Kerch, 2015). It has has been proposed that chitosan binds to the cell membrane generating H_2_O_2_ and NO in chloroplast; H_2_O_2_ activates the reactive oxygen species (ROS) scavenging system and abscisic acid (ABA) biosynthesis, while NO induces phosphatidic acid (PA) synthesis through phospholipase C (PLC) and diacylglycerol kinase (DGK) (PLC/DGK) pathways. PA enhances ABA signaling by inhibiting ABI1 (the negative regulator of ABA) whereas H_2_O_2_ stimulates jasminic acid (JA) signaling via octadecanoid pathway resulting in the up-regulation of chitosan-responsive genes (e.g., *chitinase* or *glucanase*) (Pichyangkura and Chadchawan, 2015).

## Biostimulants regulate major fruit tree physiological traits

Studies with annual plants and model species suggested that biostimulants could enhance growth, development and tolerance to abiotic stress. However, availability of this information is relative limited for fruit crops probably due to the fact that the studies with fruit trees have many disadvantages; such as the long juvenility, the large body size (require large cultivation space), the abiotic and biotic stress conditions experiencing throughout the year. Table 1 gives an overview of the available information concerning the effects of various biostimulants in fruit crops.

**Table 1:** **Functions of biostimulants in fruit tree species**.

	**Biostimulant**	**Tree species**	**Physiological effect**	**Mode of application**	**Biological origin of the biostimulant**	**References**
Fruit related and growth	Protein hydrolysate	Papaya	Increased fruit yield	Foliar spray	Acethylthioprodione and hydrolized animal skin derived aminoacids	Morales-Payan and Stall, 2003
	Protein hydrolysate	Kiwi	Stimulated shoot and root growth	Foliar spray	Protein hydrolysates from enzymatic hydrolysis of "peptone from gelatine"	Quartieri et al., 2002
	Protein hydrolysate	Passion fruit	Increased seedling growth	Foliar spray	Animal derived Protein hydrolysate	Morales-Payan and Stall, 2004
	Protein hydrolysate	Apple	Increased leaf chlorophyll and carotenoid content, enhanced photosynthesis, increase fruit yield/size	Foliar spray	Commercial extract containing zeatin, triacontanol and a commercial extract of L-cysteine and folic acid derivative	Dubravec et al., 1995
	Seaweed extract	Kiwifruit	Increased fruit weight/length and shorten maturation time	Foliar spray	*Ascophyllum nodosum* extract	Chouliaras et al., 1997
	Seaweed extract	Citrus	Increased fruit yield	Foliar spray	*Ascophyllum nodosum* extract	Fornes et al., 2002
	Seaweed extract	Olive	Increased fruit yield/oil content, increased oil linolenic and oleic acid and accelerated fruit maturation. Reduced oil palmitoleic stearic and linoleic acid	Foliar spray	*Ascophyllum nodosum* extract	Chouliaras et al., 2009
	Seaweed extract	Peanut	Increased seed protein content	Foliar spray	Commercial *Ecklonia maxima*seaweed extract (Kelpak 66)	Featonby-Smith and van Staden, 1987
	Seaweed extract	Pear	Increased fruit yield/diameter/weight and number of cells per area of parenchymatous tissue of the fruit	Foliar spray	*Ascophyllum nodosum* extract	Colavita et al., 2011
	Seaweed extract	Olive	Increased plant height, leaf number/dry weight, leaf Zn content, stem diameter	Foliar spray	Commercial seaweed extract (Sea Force)	Ibrahim, 2013
	Seaweed extract	Mango	Increased leaf area and leaf N, P, K, Mg, Zn, Fe, and Mn content. Increased fruit retention/weight,/yield/soluble sugars. Reducing vitamin C and acidity	Foliar spray	*Ascophyllum nodosum* extract	Mohamed and El-Sehrawy, 2013
	Seaweed extract	Banana	Increased fruit yield, minimized fruit moisture content and increased fruit carbohydrate, protein and mineral content	Foliar spray	Commercially *Kappapphycus alvarezii* extract	Karthikeyan and Shanmugam, 2014
	Seaweed extract	Apple	Increased fruit yield/size, increased growth of shoots and leaves, prolonged flower blooming time	Foliar spray	Commercially *Ecklonia maxima* and *Ascophyllum nodosum* extracts	Basak, 2008
	Humic substances	Apricot	Increased vegetative growth, fruit yield/size/firmness, and soluble sugar content/acidity ratio	Foliar spray and soil applications	Commercial Leonardite derived humic acid (Actasol)	Fathy et al., 2010
	Humic substances	Olive	Increased fruit yield/average size (volume), weight, shape index (length\diameter), pulp\pit ratio and moisture content. Decreased fruit acidity	Foliar spray	Commercial Leonardite derived humic acid (Actasol)	Hagagg et al., 2013
	Moringa leaf extract	Citrus	Increased leaf N, P, K, Ca, Mn and Zn. Minimized fruit drop/set. Increased yield, fruit weight/juice/soluble solid contents, vitamin C, sugars, total antioxidants and phenolic contents. Increased activity of SOD and CAT enzymes in fruit juice	Foliar spray and soil applications	*Moringa olifera* leaf extract	Nasir et al., 2016
	Pollen grain extarcts/yeast extract	Citrus	Improved fruit set/yield/weight. Reduced fruit drop and fruit acidity	Foliar spray	*Brassica napus* pollen grain extract (Milagrow) and bread yeast (*Saccharomyces cervisia)* extract	El-Boray et al., 2015
Enviromental stress and nutrition	Protein hydrolysate	Pecan	Increased nut weight, kernel weight/length/breadth. Increased fruit size and weight, increased kernel protein content and Zn, Fe, Mn, Cu foliar content	Foliar spray	Commercial organic biostimulant (Supramino) combined with urea, boric acid and zinc sulfate	Ashraf et al., 2013
	Amino acid chelate	Pear	Increased leaf Fe, Cu, Mn and Zn content	Foliar spray	Commercial aminoacid chelate foliar fertlizers (Kemito Inc.)	Koksal et al., 1999
	Seaweed extract	Citrus	Increased shoot length/dry weight, leaf area/dry weight and stem water potential. Increase plant water use efficiency. Decreased leaf photosynthesis/stomatal	Soil drench or foliar application	Commercial *Ascophyllum nodosum* extract (Stimplex)	Little and Spann, 2010
	Seaweed extract	Citrus	Increased growth and stem water potential	Soil drench or foliar application	Brown *Ascophyllum nodosum* seaweed extract	Spann and Little, 2011
	Seaweed extract	Almond	Increased shoot leaf area, shoot length and biomass	Foliar spray	Mixture of commercial plant based biostimulants (MegaFol, Brexil-Zn, and MC-Extra) and commercial microbial fermentation product derived from a proprietary mix of organic cereal grains (GroZyme)	Saa et al., 2015
	Humic substances	Apricot	Increased tree yield, vegetative growth, total leaf chlorophyll and leaf N, P, K, Mg content	Foliar spray	Commercial Leonardite derived humic acid (Actosol) and yeast (*Saccharomyces cerevisiae*) extract	Fatma et al., 2015
	Leonardite extract	Olive	Increased shoot growth, and leaf K, B, Mg, Ca and Fe content	Foliar spray	Leonardite derived humic acid	Fernández-Escobar et al., 1996
Tree physiology	Protein hydrolysate	Banana	Provoked early flowering; increased leaf chlorophyll and proline content. Reduced fruit sugars, proteins, amino acids, phenolics and flavonoids	Foliar spray and soil applications	Chicken feather derived Protein hydrolysate	Gurav and Jadhav, 2013
	Protein hydrolysate	Olive	Increased pollen tube elongation	Foliar spray	Animal derived Protein hydrolysate (Siapton)	Viti et al., 1990

### Foliar application of biostimulants promote fruit-related growth characteristic

Protein hydrolysates receive attention as biostimulants in fruit science. It has been demonstrated that commercial mixtures of protein hydrolysates enhanced the overall yield in papaya (Morales-Payan and Stall, 2003) and apple (Dubravec et al., 1995). Quartieri et al. (2002) studied the effect of foliar application of animal-derived protein hydrolysates obtained by different rates of protein hydrolysis at kiwi plants. These authors reported that the fraction with the lowest molecular weight and at low doses stimulated shoot and root biomass, while the fraction with high molecular weight was able to promote shoot growth. In banana tree, feather-derived protein hydrolysates minimized the harvesting date by 28 days and enhanced the number of hands per brunch, fingers per hand and brunch. Although the exact mechanism remains unknown, this is likely to occur through higher chlorophyll and reduced sugar contents (Morales-Payan and Stall, 2004).

Several commercial and experimental seaweed extracts can also be employed to produce potent biostimulants. Foliar application of *A. nodosum* extract in kiwi plants after flowering increased the weight and maturity of the harvested fruits (Chouliaras et al., 1997). In clementine and orange trees, foliar spray of seaweed extracts at budding stage positively affected bud sprouting and full bloom, and enhanced gibberellin content and fruit yield (Fornes et al., 2002). Apple trees treated with seaweed extract exerted an improved flowering, vegetative growth and yield (Basak, 2008). Treatment of seaweed extracts to olive plants before bloom improved oil quality characteristics (Chouliaras et al., 2009) as well as mineral content, leaf dry weight and stem diameter (Zulaikha, 2013). Application of seaweed extract to peanut leaves enhanced seed yield and increased the protein content of the harvested seed (Featonby-Smith and van Staden, 1987). In the work of Colavita et al. (2011), foliar application of seaweed extract in pear tree increased fruit diameter, fruit weight and number of cell per area of parenchymatous tissue. Additionally, seaweed extracts have shown promising results as growth-yield-promoting agents in tropical trees (Mohamed and El-Sehrawy, 2013; Karthikeyan and Shanmugam, 2014). Experiments with citrus plants showed enhanced yield and fruit quality (mineral status/acidity) following foliar application of moringa leaf or pollen grain extract/yeast extracts (El-Boray et al., 2015; Nasir et al., 2016).

### Biostimulants enhanced tolerance to environmental stress and improved nutrient availability

Another well-known physiological action of biostimulants is their ability to induce tree tolerance against environmental stress. Hamlin' sweet orange trees exposed to commercial extract of brown seaweed displayed tolerance toward drought (Spann and Little, 2011). In another study, citrus plants sprayed with seaweed extract, under drought conditions, showed intermediate water use efficiency (Little and Spann, 2010). The overall results suggested that seaweed extract promotes stem water potential in citrus rootstocks under full irrigation and drought, as well as affects photosynthesis, stomatal conductance and water use efficiency in a cultivar-dependent manner (Little and Spann, 2010).

Enhanced fruit tree growth and yield by biostimulants have been accompanied in some cases by improved nutrient uptake. For example, pear (cv. Williams) leaves treated with amino acid chelate showed higher leaf Fe and Zn content (Koksal et al., 1999). Pecan trees treated with protein hydrolysates extract increased Zn, Fe, Mn, and Cu foliar content (Ashraf et al., 2013). Leave-applied humic substances in apricot and olive trees stimulated mineral level (N, P, K, and Mg) (Fatma et al., 2015) while seaweed extracts improved shoot growth and leaf area upon nutrient deprivation in almond tree (Saa et al., 2015). In addition, leonardite extract enhanced K, B, Mg, Ca, and Fe accumulation in olive leaves (Fernández-Escobar et al., 1996), indicating that humic substances may affect nutrient leaf content through mechanisms other than the direct formation of complexes and chelates in the soil. The plant hormone like activity attributable to humic substances (Mora et al., 2014) is probably the main biological factor responsible for the positive effects exerted by humic substances on fruit tree physiology.

### Biostimulants influenced specific features of fruit tree physiology

In addition to the above well-established role of biostimulants, some interesting data revealed that biostimulants are able to regulate specific physiological features in fruit trees. For example, their application has been correlated with increased biosynthesis of antioxidant-related compounds. Chicken feather-derived protein hydrolysate applied in banana plants at flower induction period enhanced the accumulation of several bioactive substances, like amino acids, phenolics, and flavonoids (Gurav and Jadhav, 2013). Finally, Viti et al. (1990) demonstrated the positive effect of foliar application of a commercial protein hydrolysate upon *in vivo* and *in vitro* pollen tube elongation in olive plants.

## H_2_O_2_ and NO priming dynamic in fruit tree physiology

**Figure 1:**
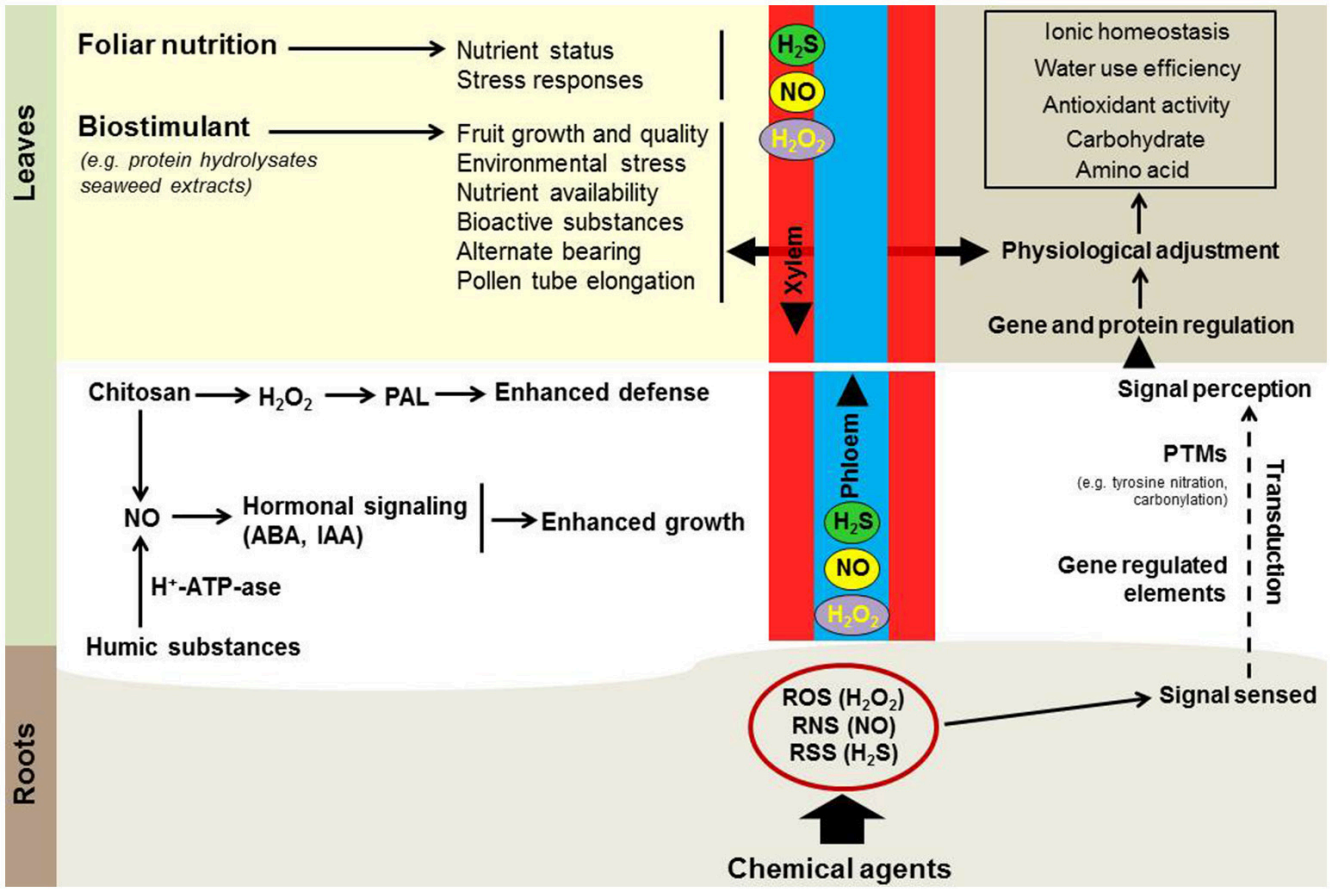
**Model of likely biostimulant interaction with H_**2**_O_**2**_, NO, and H_**2**_S priming signaling in fruit trees (see text for details)**.

Reactive oxygen (e.g., H_2_O_2_), nitrogen (e.g., NO), and sulfur (e.g., H_2_S) molecules are currently recognized as important signaling species involved in stress acclimation. So far, only few studies provide convincing data for the induction of a primed state of fruit trees in the context of environmental stress. Experimental evidence on H_2_O_2_, NO, or H_2_S root-treated Citrus aurantium plants demonstrated that these chemical treatments enhance acclimation to salinity and drought (Molassiotis et al., 2016). As a general conclusion, H_2_O_2_ and NO chemical treatments altered many proteins involved in photosynthesis process (e.g., Rubisco activase, phosphoglyceratekinase, glyceraldehyde-3-phosphate dehydrogenase, phosphoribulokinase, carbonicanhydrase) and regulated ROS/RNS-based posttranslational protein modifications (PTMs), such as protein carbonylation, *S*-nitrosylation, tyrosine (Tyr) nitration, thereby altering leaf protein function and activity (Tanou et al., 2009, 2012a). In another report, it was demonstrated recently that NaHS initially triggers a signaling stream in leaves where the level of nitrite, NOx, *S*-nitrosoglutahione reductase, and the expression of genes involved in NO-generation (eg., NR, NiR, NOS-like, NADHox, NADHde, AOX) along with the expression of genes involved in ABA biosynthesis (eg., 9-*cis*-epoxycarotenoiddioxygenase), play a pivotal role in citrus acclimation to drought stress (Ziogas et al., 2015; Figure 1).

Apart from H_2_O_2_ and NO, there are experimental data, although not extensive, indicating that melatonin (Mel) plays signaling roles in several physiological process of trees species. For instance, root-applied Mel prevented NaCl-associated toxicity symptoms in leaves of *C. aurantium* seedlings and regulated the expression of an osmoregulated gene (*MIPS*), an anion-associated channel (*SLAH1*), and a salt-response transcription factor (*MYB73*), indicating that sugar metabolism, ion homeostasis and transcription regulation were triggered by Mel (Kostopoulou et al., 2015). Also, Mel treatment activates H_2_O_2_-scavenging enzymes, which might be a part of the mechanism implicated in the delay of senescence in peach fruit (Gao et al., 2016). However, a limited number of data have been obtained with Mel application in leaves of fruit trees. In a particular interesting paper, exogenous Mel delayed dark-induced senescence in apple leaves through the enhancement of some ROS scavenging enzyme activities, which contributed to the elimination of the H_2_O_2_ excess generated in stressed leaves, while maintained the ascorbic acid and glutathione content higher than in control leaves (Wang et al., 2012). Future work is required to identify novel roles for Mel and their interaction with other ROS signals in fruit tree physiology.

## Interplay of biostimulants with H_2_O_2_ and NO priming

A body of evidence supports the existence of a link between biostimulants and H_2_O_2_/NO-based priming in several metabolic processes. An example is that chitosan application in plant tissues triggers an H_2_O_2_–originated oxidative burst (Zhao et al., 2007) that orchestrates the induction of plant defense enzymes, including phenylalanine ammonia-lyase (PAL), a key enzyme in phenolics biosynthesis. Increased PAL activity and overproduction of phenolic compounds following chitosan treatment has been reported in several fruit trees, like papaya (Ali et al., 2012), litchi (Zhang and Quantick, 1997), apricot (Ghasemnezhad and Shiri, 2010), and loquat (Ghasemnezhad et al., 2011). Along with H_2_O_2_, chitosan is also involved in the stimulation of NO in plant tissues. It was proposed that exogenous application of oligochitosan resulted in NO generation in chloroplast, nucleus and finally in the whole cell (Zhang et al., 2011). The production of NO after chitosan application interacts with ABA negative regulator ABI1, leading to ABA signaling and massive gene modulation (Zhang et al., 2004). It has been reported that humic substances induced plant growth by apoplastic acidifying via cross activation of plasma membrane H^+^-ATP-ase and NO production (Zandonadi et al., 2010). Similarly, the promotion of shoot growth by humic substances can occur through both IAA- and NO-dependent pathways (Mora et al., 2014). Upon this type of plant morphological change, the interplay of humic substances and NO has been proposed (Figure 1).

## Challenges and opportunities

The examples presented in this review documented the potential of foliar-based nutrition and biostimulants to enhance fruit tree performance; however, there are insufficient experimental data on how these treatments affect cell metabolism. Within this context, the application of art systems-biology metadata approaches, based on global analysis of transcriptomes, proteomes and metabolomes together with bioinformatic platforms would substantially contribute to reach this goal. Another interesting area that would be investigated is whether, as in root treatments with priming molecules, leaf-applied biostimulants act as priming elicitors at the whole-plant level, thereby systemically sensed by roots. A model explaining the mode of priming action of H_2_O_2_ and NO against salinity has been recently proposed by Molassiotis et al. (2016): Using citrus seedlings, the authors showed that phloem is the likely path for self-propagated systemic transmission or movement of H_2_O_2_ and NO signals from root to leaves. Another field where information is lacking is whether and how foliar nutrition and biostimulants could act synergistically, for example, upon the same type of stress or between biotic and abiotic stresses. Analogously, a scientific area for future research is the combination among some of the various biostimulants presented in this review. It is interesting also to note that a large number of studies are focused on young fruit trees. Thus, it is important to monitor and observe the foliar nutrition/biostimulants effects at the level of an orchard ecosystem, specifically in view of the ever-increasing applications of these materials. Upcoming investigations are also expected to characterize the application method and standardization of treatments for cost-effective fruit protection strategies. Furthermore, the application of both biostimulants and chemical priming agents in young fruit trees in nursery or at the planting stage could be anticipated because it would reduce the costs associated with later cultivation treatments in the orchard. Despite these open questions, the technology of foliar nutrition and biostimulants might be combined with all available modern agronomic practices, such as precision agriculture and innovative decision-making systems, to create novel approaches in fruit tree cultivation.

## Author contributions

GT, VZ and AM wrote the paper.

### Conflict of interest statement

The authors declare that the research was conducted in the absence of any commercial or financial relationships that could be construed as a potential conflict of interest.
